# Death from Hemorrhage Caused by Lancing Gums

**Published:** 1899-11

**Authors:** B. H. Catching

**Affiliations:** Atlanta, Ga.


### Domestic Correspondence.

### DEATH FROM HEMORRHAGE CAUSED BY LANCING GUMS.

To the Editor:

Sir,—News has just reached me of the death of a very strong, robust child resulting from hemorrhage caused by lancing the gums to aid tooth eruption.

This accident will be the cause of untold suffering to little ones, wherever this case is reported. Fond parents will dread the lancet, not thinking or knowing of the great benefit of this instrument when judiciously used, but fearing a like result. I fancy that it is an instrument too little used during the process of teething. In my own hands it has been of untold benefit, and never any harm. I must add another incident during teething, which occurred a few months since. A child by some means dislodged a loose temporary tooth, and died from the hemorrhage therefrom. He was a "bleeder," evidently. The question occurs to me, Would not the administration of a few grains of tannic acid have proved effectual in each case?

I am glad to announce that, through the untiring efforts of Dr. H. H. Johnson, of Macon, the office of Dental Surgeon to the Georgia Insane Asylum has been created. Dr. Edward Tignor, of Atlanta, has been elected by the trustees to fill this important position. He has entered upon this important work, and much good may be expected from his wise efforts in the amelioration of the suffering of the inmates of this noble institution.

It is a position that every institution of the kind should have filled. Dr. Johnson has made a close study of the reflex action of diseased and malposed teeth, and set to work years ago to accomplish that which has just been done. Too much credit cannot be given him for the accomplishment of his great purpose. Dr. Tignor's work will be watched with much interest; nothing but good can come of it.

B. H. Catching.

Atlanta, Ga.

